# Combining Concepts Across Categorical Domains: A Linking Role of the Precuneus

**DOI:** 10.1162/nol_a_00039

**Published:** 2021-07-13

**Authors:** Giuseppe Rabini, Silvia Ubaldi, Scott Fairhall

**Affiliations:** Centre for Mind/Brain Sciences (CIMeC), University of Trento, Trento, Italy

**Keywords:** fMRI, combinatorial semantics, language, sentences, precuneus, category selectivity

## Abstract

The human capacity for semantic knowledge entails not only the representation of single concepts but also the capacity to combine these concepts into the increasingly complex ideas that underlie human thought. This process involves not only the combination of concepts from within the same semantic category but also frequently the conceptual combination across semantic domains. In this fMRI study (*N* = 24) we investigate the cortical mechanisms underlying our ability to combine concepts across different semantic domains. Using five different semantic domains (People, Places, Food, Objects, and Animals), we present sentences depicting concepts drawn from a single semantic domain as well as sentences that combine concepts from two of these domains. Contrasting single-category and combined-category sentences reveals that the precuneus is more active when concepts from different domains have to be combined. At the same time, we observe that distributed category selectivity representations persist when higher-order meaning involves the combination of categories and that this category-selective response is captured by the combination of the single categories composing the sentence. Collectively, these results suggest that the precuneus plays a role in the combination of concepts across different semantic domains, potentially functioning to link together category-selective representations distributed across the cortex.

## INTRODUCTION

The human capacity for semantic knowledge involves not only the representation of single concepts but also the capacity to combine these concepts into the increasingly complex ideas that underlie human thought. A wealth of research on single concepts has shown that the human brain implements semantic representation over a complex system involving regions that are sensitive to specific semantic classes of objects, such as people, food, or places, in addition to regions that are generally more active for semantically richer stimuli, regardless of category. Accordingly, the functioning of the semantic system is reflected in a dynamic interplay between domain-specific and domain-general representations (Binder, 2016; Binder et al., 2009; Binder & Desai, 2011; Chen et al., 2017; Kiefer & Pulvermüller, 2012; Martin, 2016; Patterson et al., 2007). The distributed representation of semantic knowledge in the brain potentially indicates a fundamental organisational principle, whereby basic object-related knowledge extends to complex, multi-faceted units of meaning.

The combination of concepts into higher-order representations not only involves the linking of concepts from within the same semantic domain but also frequently entails the flexible association of concepts spanning different domains. For instance, reading about a boy playing with his dog in the garden requires the system to link concepts from distinct conceptual domains (i.e., people, animals, and places) to build a distinct and coherent representation. To date, however, it is still unclear how category-selective brain regions interact when concepts from different domains have to be combined into higher-order semantic units. In particular, there remain two open questions: (1) Do specific brain regions coordinate information contained in category-selective cortical regions? And (2) do complex ideas that combine category information from multiple domains continue to utilise those domain-selective representations of single concepts, or do derived multi-categorical concepts rely more on domain-general semantic mechanisms?

The general semantic processing of semantically richer stimuli recruits a left-lateralised cortical network encompassing several heteromodal associative regions (the angular gyrus (AG), lateral temporal cortex, ventral temporal cortex, dorso-medial and ventro-medial prefrontal cortex, inferior frontal gyrus (IFG), and the precuneus) (Binder et al., 2009). Although modality-specific activations emerge during modality-specific conceptual processing—for example, perceptual/motor-related concepts activate the respective perceptual/motor brain areas (see Binder & Desai, 2011; Borghesani & Piazza, 2017; Kiefer & Pulvermüller, 2012)—the general semantic network appears to be clearly distinguished from primary sensory and motor cortices (Binder et al., 2009). While a strong embodied view of cognition states that conceptual knowledge emerges exclusively from sensory and action/motor experience and is therefore grounded and represented in the related cortical regions (Barsalou, 2010; Gallese, 2005; see also Mahon & Caramazza, 2008, for a critical perspective), a softer version (“embodied abstraction”; Binder & Desai, 2011) gives an alternative view. Specifically, it states that different levels of abstraction starting from sensory, motor, and emotional experiences model our conceptual representation. Higher-level concepts can be abstracted from the primary sensory meaning, and different levels of abstraction can be selectively activated depending on several factors, such as context and task demand. In this view, heteromodal cortices might be involved in the representation of more abstract—high-level—concepts, which are not necessarily and directly linked to sensorimotor experience.

The position has found complementary support in neurocomputational models of neural semantic representation (Chen et al., 2017) and in recent theoretical proposals (Lambon Ralph et al., 2016). In the controlled semantic cognition framework, the representational subsystem has been described as “the hub-and-spoke model” (Patterson et al., 2007; Rogers et al., 2004). This model predicts that concepts emerge from both verbal and nonverbal experience and that modality-selective cortices, distributed across the whole brain, represent this specific information (“the spokes”). Furthermore, a unique *transmodal* core region (“the hub”), identified in the ventro-lateral anterior temporal lobe (ATL) (see also Visser et al., 2010), would be engaged in modality-invariant representations, so that this region can represent object-concepts in multimodal, more abstract, global units. Recent advances further elaborated on this proposal by advocating a graded specialisation within the ATL, based on the different connectivity patterns of its subparts (Lambon Ralph et al., 2016; Rice et al., 2015).

The presence of distributed cortical representations of semantic information, linked to the notion of “spokes,” as previously defined, grounds its evidence in longstanding research focusing on object–concept representation in the brain and how the semantic category to which the object belongs can affect its cortical representation. There is now compelling evidence that conceptual knowledge can be selectively impaired following focal brain lesions (Capitani et al., 2003; Caramazza & Mahon, 2003; Miceli et al., 2000; Warrington & Shallice, 1984) and that the neural responses of discrete brain regions are more sensitive to specific semantic categories (Caramazza & Shelton, 1998; Kuhnke et al., 2020; Mahon et al., 2009; Mahon & Caramazza, 2011; Martin, 2016; Noppeney et al., 2006). This cortical category selectivity, at least for people and places, has been shown to persist, both considering specific, unique entities (people: “Leonardo DiCaprio”; places: “The colosseum”; Fairhall et al., 2014) and general semantic knowledge (“kind of”—people: “lawyer”; places: “courthouse”; Fairhall & Caramazza, 2013a), thus demonstrating an additional category-specific sensitivity for concepts abstracted from their principal sensorimotor counterparts. Like object category, accessed content also selectively recruits specialised conceptual representations. When atypical information is accessed about people or food, such as the geographical provenance of the item, regions classically associated with place selectivity are recruited (Fairhall, 2020).

Semantic category sensitivity in the human brain has also been highlighted through data-driven approaches, by assessing brain responses to naturally spoken narrative stories (Deniz et al., 2019; Huth et al., 2016), further mapping single word-related semantic selectivity on the whole brain surface (see also Pereira et al., 2018).

While crucial for the understanding of conceptual knowledge, single concepts do not capture the complexities of the semantic contents we manage in everyday life (Frankland & Greene, 2020). The capacity to flexibly combine multiple concepts from distinct categories into unitary representations is a fundamental feature of human semantic cognition. The present study focuses on the cortical mechanism underlying the formation of higher-order meaning that necessitates the combination of concepts belonging to different semantic domains, specifically those concepts that are frequently represented across distinct category-selective brain regions. To this end, we implemented an event-related fMRI paradigm presenting written sentences regarding a single semantic category (i.e., People, Places, Food, Objects, and Animals) or sentences encompassing two distinct conceptual domains (e.g., People and Food). Our objective was to answer two main questions: (1) Are brain regions differentially activated when information from different semantic categories has to be combined? (2) Do higher-order semantic representations that combine concepts across category continue to rely on category-specific representations, or do these more derived combinatorial semantic meanings rely more heavily on general semantic representations?

## MATERIALS AND METHODS

### Participants

Twenty-eight native Italian speakers were recruited for the study. Three participants were excluded due to head motion exceeding 2 mm during scanning. Another participant did not perform the entire protocol and therefore was excluded. Thus, the final sample consisted of 24 participants (12 males, mean age 24.9 years). Before entering the scanner, participants underwent a medical interview with a neurologist, and all of them reported no history of neurological or psychiatric disease. Participants gave informed consent and were compensated for participation (15 €/hour). The study was conducted in line with the declaration of Helsinki (1964, amended in 2013) and was approved by the Ethical Committee of the University of Trento.

### Experimental Design

#### Stimuli

The stimuli set was composed of 288 Italian written sentences formed by a subject, a verb, and a complement. Sentences were of three types. (i) *Single-category sentences* (mean number of words: 5.79 (0.67)): sentences in which both the subject and the complement belong to the same semantic category [People, Places, Food, Objects, Animals]. (ii) *Combined-category sentences* (mean number of words: 5.59 (0.60)): sentences in which the subject and the complement belong to different semantic categories. There were 10 combinations of the five main conceptual categories [People & Places, People & Food, People & Objects, People & Animals, Places & Food, Places & Objects, Places & Animals, Food & Objects, Food & Animals, Objects & Animals]. (iii) *Bizarre sentences* (mean number of words: 5.6 (0.68)): grammatically correct sentences but with an anomalous semantic meaning (e.g., “The window was inside the tomato”). Representative sentences (and the English translation) for each sentence type are presented in Table 1 (the full list can be found in Supplementary Table S3; supporting information can be found online at https://www.mitpressjournals.org/doi/suppl/10.1162/nol_a_00039). There were a total of 16 sentences for each of the single- and combined-category conditions, and 48 sentences for the bizarre condition.

**Table 1.** Representative stimuli

	Sentence category	Representative sentence
**Single**	People	I poliziotti arrestano i ladri
		*The cops arrest the thieves*
	Places	Le fabbriche sono fuori dalle cittá
		*Factories are outside cities*
	Food	I bigné sono ripieni di cioccolata
		*Cream puffs are filled with chocolate*
	Objects	Le lampade illuminano i tavoli
		*Lamps illuminate tables*
	Animals	I cani rincorrono le lepri
		*Dogs chase hares*
**Combined**	People & Places	Gli studenti si trovano all’universitá
		*Students are at the university*
	People & Food	Le mamme cuociono le crostate
		*Mothers bake pies*
	People & Objects	I camerieri sistemano le forchette
		*Waiters arrange forks*
	People & Animals	I cacciatori sparano ai cervi
		*Hunters shoot deer*
	Places & Food	Le pere crescono nel frutteto
		*Pears grow in the orchard*
	Places & Objects	Le forbici si vendono al supermercato
		*Scissors are sold at the supermarket*
	Places & Animals	I delfini saltano nel mare
		*Dolphins jump in the sea*
	Food & Objects	I caffé si bevono nelle tazzine
		*Coffees are drunk in cups*
	Food & Animals	I gatti bevono sempre il latte
		*Cats always drink milk*
	Objects & Animals	I topi evitano le trappole
		*Mice avoid traps*
**Bizarre**		La finestra era dentro al pomodoro
		*Window was inside the tomato*

To assess the imageability of different sentences, 10 participants (who did not take part in the main experiment) rated the imageability of each sentence on a 5-point Likert scale. Globally, the sentences were perceived as highly imaginable (mean = 4.17). Imageability ratings were averaged within each condition and statistical analysis performed across participants. A paired sample *t* test between the average ratings for single-category (4.105) and combined-category sentences (4.197), revealed a subtle difference (0.092, *t*(9) = 2.02, *p* = 0.0371). Comparatively, variation across sentence types was more pronounced over conditions within the 5 single-category conditions (range: 3.969–4.375) and within the 10 combined-category conditions (range: 3.769–4.456).

Sentences were matched on the proportion of action to state verbs in the single (71%) and combined category sentences (80%, *z* = 1.6, *p* = 0.12; normal approximation to the binomial). We additionally considered the sociality of verbs. Verbal phrases were presented in isolation and labelled by two raters (S.U. and G.R.) according to the criteria: “likely to relate to an interaction between two individuals.” Social verbs occurred more frequently in single-category sentences (15%) compared to combined-category sentences (5.6%). This was primarily driven by an over-representation of social verbs in the person–person condition, where they were present in 12 out of 16 sentences.

#### fMRI experimental task

The fMRI session was divided into six experimental runs. In each run, there were 8 trials for each single- and combined-category sentence (5 single and 10 combined), 24 trials with bizarre sentences, and an additional 24 fixation-cross null-events. In an event-related paradigm, sentences were pseudo-randomized across runs and randomly interleaved with fixation cross events (sentences were repeated three times across the experiment). Using MATLAB (www.mathworks.com) and Psychophysics Toolbox Version 3 (psychtoolbox.org), each sentence was presented in black font against a gray background and presented consecutively in three fragments (subject, verb, complement). Each trial lasted 2.5 s. Each sentence-fragment was presented consecutively on the centre of the screen for 400 ms. After the 1.2 s of stimulus presentation, a black fixation cross appeared in the centre of the screen for the remainder of the trial. The participant’s task was to indicate via button-press if the sentence was semantically meaningful (index finger) or was “bizarre” (right middle). Reaction times (RTs) were calculated from the onset of the last sentence fragment, and responses faster than 400 ms or slower than 1,700 ms were excluded. RT data for one participant was unavailable due to measurement error.

#### Post-scanner test

After the fMRI session, participants were again presented the meaningful sentences they had read in the fMRI session. In the task, a part of the sentence (subject/complement) was missing, and participants were instructed to complete the missing part.

### MRI Scanning Parameters

Functional and structural data were collected with a Prisma 3T scanner (Siemens AG, Erlangen, Germany) at the Centre for Mind/Brain Sciences (CIMeC) of the University of Trento. Participants lay in the scanner and viewed the visual stimuli through a mirror system connected to a 42″, MR-compatible Nordic NeuroLab LCD monitor positioned at the back of the magnet bore. Data collection was performed using a 64-channel head coil. Functional images were acquired using echo planar imaging (EPI) T2*-weighted scans. Acquisition parameters were: repetition time (TR) of 2 s, an echo time (TE) of 28 ms, a flip angle of 75°, a field of view (FoV) of 100 mm, and a matrix size of 100 × 100. Total functional acquisition consisted of 1,266 volumes for the six experimental runs, each of 78 axial slices (which covered the whole brain) with a thickness of 2 mm and a gap of 2 mm, AC/PC aligned. High-resolution (1 × 1 × 1 × mm) T1-weighted MPRAGE sequences were also collected (sagittal slice orientation, centric phase encoding, image matrix = 288 × 288, FoV = 288 mm, 208 slices with 1-mm thickness, TR = 2,290, TE = 2.74, inversion time (TI) = 950 ms, 12° flip angle).

### fMRI Data Analysis

Data were analysed and preprocessed with SPM12 (https://www.fil.ion.ucl.ac.uk/spm/). The first four volumes of each run were dummy scans. All images were corrected for head movement. Functional images were normalized to the Montreal Neurological Institute (MNI) T1 space, resampled to a voxel size of 2 × 2 × 2 mm and spatially smoothed with 6-mm FWHM kernel. Subject-specific parameter estimates (β weights) for each of the 16 conditions (see Experimental Design section for details) were derived through a general linear model (GLM) and a more lenient implicit mask for inclusion in the GLM (0.1 instead of the SPM default of 0.8). The control condition with a fixation cross formed the implicit baseline. The six head-motion parameters were included as additional regressors of no interest.

### Region of Interest Selection

Region of interest (ROI) analysis was performed within category-selective ROIs defined using an Omnibus ANOVA to highlight cortical regions showing a differential response across categories for the single category sentences only. ROIs were defined as the intersection between a sphere of 5-mm radius around the group peak coordinates, and the activation map for the Omnibus ANOVA thresholded at *p* < 0.001. The location of ROIs is indicated in Supplementary Table S1.

### Additional Voxel-Wise Multivariate Pattern Analysis

A supplementary voxel-wise multivariate pattern analysis (MVPA) was performed within a precuneus ROI showing a greater response to combined-category than single-category sentences. In this analysis, the constituent single-category sentences were used to predict patterns produced by combined-category sentences. Specifically, correlation based MVPA analysis was performed between pairs of combined category sentences (e.g., (A) people and food sentences versus (B) place and object sentences) using the summed pattern of responses of the relevant single category sentences (e.g., (C) people+food single category sentences and (D) place+object single-category sentences). To assess whether category information present for single-category sentences persisted in the combined-category sentences, correlations between unlike sentence types (A & D; B & C) were subtracted from like sentence types (A & C; B & D). This process was repeated 45 times for each of the possible pairwise combinations of the ten combined-category conditions. One-sample *t* tests were performed on the resulting values to allow inference.

## RESULTS

### Behavioural Results

Reaction times on the meaningfulness judgment did not differ between single-category (mean = 716 ms, *SD* = 120) and combined-category (mean = 723 ms, *SD* = 120) sentences (*t* < 1). A repeated-measure ANOVA revealed that RTs differed among sentence-category for single-category sentences (*F*(4, 92) = 17.2, *p* < 0.001), and combined-category sentences (*F*(5.3, 121) = 10.5, *p* < 0.001; Greenhouse-Geisser corrected). In this way, RT-related effects will not influence comparisons between single-category and combined-category sentences but may influence category-selective effects. This will be further discussed in the relevant sections.

Task compliance was high, with meaningful sentences being judged meaningful 90.5% of the time and bizarre sentences identified as bizarre 84.2% (16.51) of the time. In the post-scanner test, participants were able to provide the missing sentence fragment with a high degree of accuracy (mean = 68.56%, *SD* = 15), further indicating a high level of engagement in the scanner task.

### Combination of Concepts Across Semantic Domains

Our first goal was to determine which brain regions may coordinate the combination of concepts across different semantic domains. To identify brain regions showing an increased fMRI response when conceptual domains are combined, we compared sentences presenting a combination of conceptual categories (e.g., Places & Animals: “The dog is in the kitchen”) to sentences involving a single conceptual domain (e.g., Animals: “The cat is next to the dog”).

**Figure 1.**
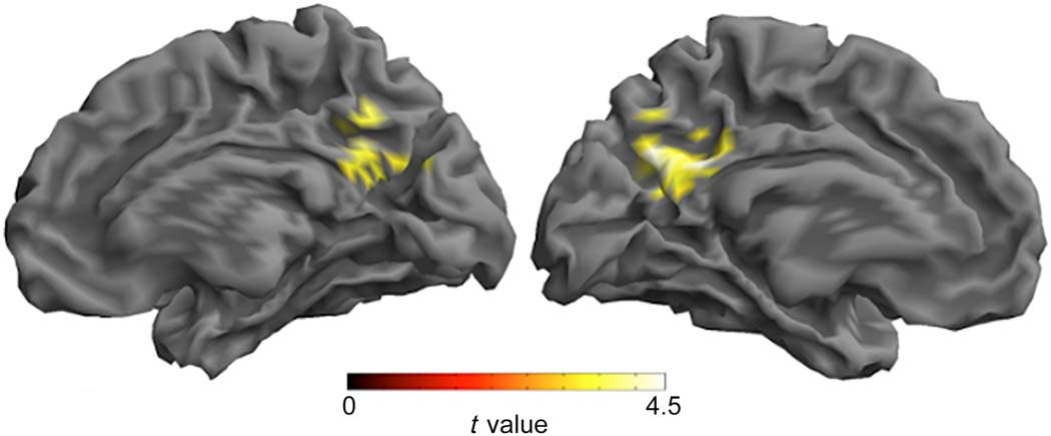
Selective activation related to combinations of semantic concepts compared to concepts related to a single semantic category. The whole-brain univariate response for the contrast [Combined-Category sentences > Single-Category sentences] is shown in the figure. A significant cluster emerged in the precuneus (Extent 1,214 voxels, *p* < 0.001_fwe-cluster_, peak = [−4 −50 28]).

The weighted contrast [Combined-Category sentences > Single-Category sentences] identified a significant cluster in the precuneus (Extent 1,214 voxels, *p* < 0.001_fwe-cluster_, peak = [−4 −50 28], Figure 1), indicating that the precuneus plays a role in the combination of concepts across categorical domains. To exclude the possibility that a single category was driving this effect, we repeated the analysis iteratively, excluding one category from both the single-category and combined-category sentences. The increased response in the precuneus for sentences that combine information across categories persisted when removing animals (Extent: 1,189 voxels, *p* < 0.001_fwe-cluster_, peak = [2 −54 18]), people (Extent: 210 voxels, *p* = 0.002_fwe-cluster_, peak = [−6 −48 28]), places (Extent: 415 voxels, *p* < 0.001_fwe-cluster_, peak = [−12 −56 20]), food (Extent: 219 voxels, *p* = 0.002_fwe-cluster_, peak = [10 −54 40]) and objects (Extent: 1,525 voxels, *p* < 0.001_fwe-cluster_, peak = [−4 −68 32]).

This iterative leave-one-category-out process was also used to assess the potential role of imageability on activation within the precuneus by recalculating single-/combined-category differences in imageability based only on the included conditions. On iterations where imageability was balanced between single-category and combined-category conditions, when removing Places (difference = 0.02, *t* < 1) and Objects (difference = 0.04, *t* < 1), the greater response in the precuneus for combined-category sentences is seen to persist, indicating that imageability does not drive this effect. We performed a second analysis to assess which brain regions are modulated by sentence imageability over our 15 stimulus types. Using a weighted contrast, we identified voxels where the response amplitude was predicted by the average imageability rating of the 15 experimental conditions. While we observed positive evidence for response modulation by imageability in left ATL (−54*x* −8*y* −14*z*; extent: 148 voxels; *p* = 0.017, cluster-corrected) and vmPFC (−4*x* 58*y* −10*z*; extent: 171 voxels; *p* = 0.008, cluster-corrected), evidence was not present in the precuneus. Collectively, these results suggest that, in the present paradigm, imageability-related processes are not driving the increased fMRI response in the precuneus for combined-category compared to single-category sentences. Like nouns, the sociality of verbs may selectively influence cortical activation and is known to increase the response in the precuneus (Lin et al., 2019). As social verbs are more prevalent in the single-category condition (see Materials and Methods), such factors cannot account for the increased response for combined-category sentences.

### Category Selective Semantic Representations

**Figure 2.**
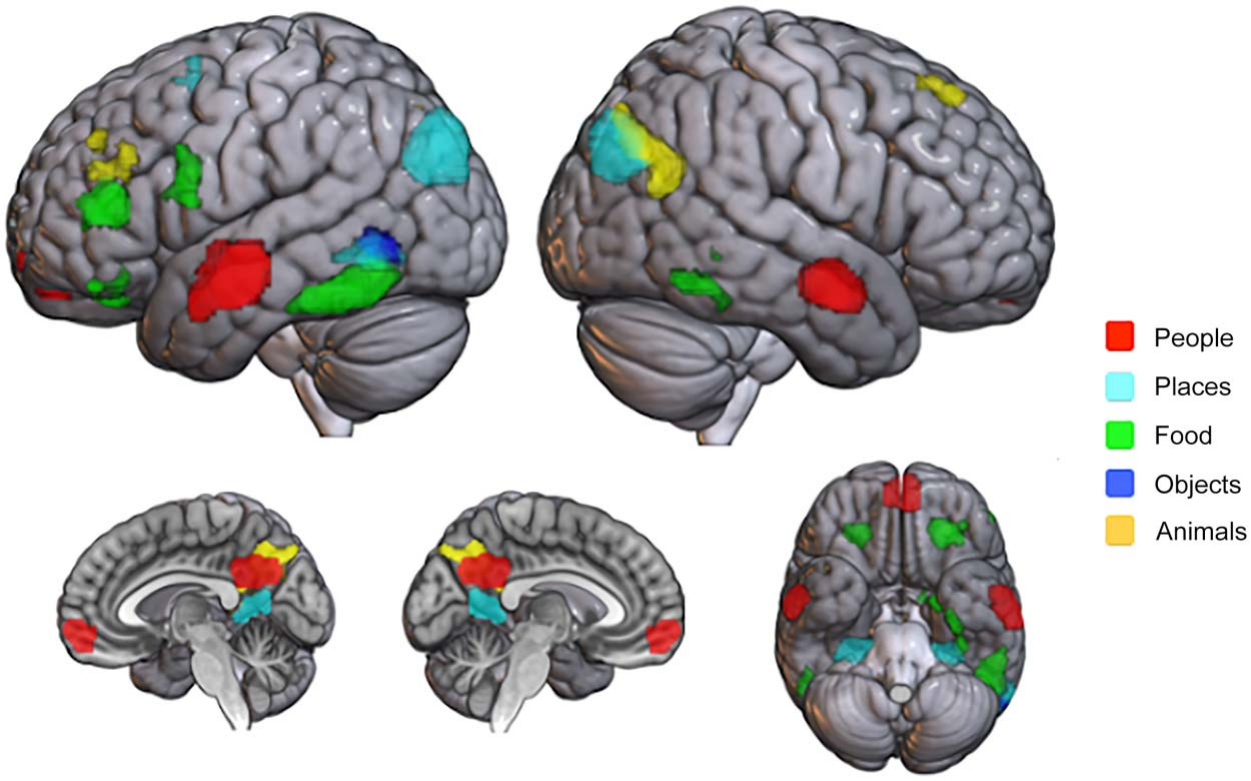
Category-sensitive activations associated with single-category (*People*, red; *Places*, light blue; *Food*, green; *Objects*, blue; *Animals*, yellow) sentences. Significant clusters (voxel-wise *p* < 0.001, uncorrected, FWE cluster-corrected *p* < 0.05) are shown on the same brain surface map (standard MNI152, MRIcroGL software, https://www.nitrc.org/projects/mricrogl). Transparency has been applied to highlight the whole identified clusters. We reported significant clusters for People in the vmPFC, precuneus, and bilateral ATL; for Places in bilateral PPA, bilateral TOS, bilateral RSC, left MTG, left aSTG, and left dSFG; for Food in the bilateral OFC, left IFG, left preMotor, left pITG, left amygdala, left VTC, and right pMTG; for Objects in the left MTG; and for Animals in the precuneus, right SFG, left dlPFC, left IPS, and right TPJ.

To characterise category-selective semantic responses, we contrasted each single category with the remaining four single categories (e.g., People > [Places, Food, Objects, Animals], see Figure 2). We observed strong category-selective fMRI responses for People in ventro-medial prefrontal cortex (vmPFC), precuneus, and bilateral ATL; for Places in bilateral para-hippocampal place area (PPA), bilateral tranverse occipital sulcus (TOS), bilateral retrosplenial complex (RSC), left middle temporal gyrus (pMTG), left anterior superior temporal gyrus (aSTG), and left dorsal superior frontal gyrus (dSFG); for Food in the bilateral orbito-frontal cortex (OFC), left IFG, left preMotor cortex, left and right posterior inferior temporal gyrus (pITG), left amygdala, left ventro-temporal cortex (VTC), and right pITG; for Objects in the left inferior temporal gyrus (ITG); for Animals in the precuneus, right superior frontal gyrus (SFG), left dorso-lateral prefrontal cortex (dlPFC), left inferior parietal sulcus (IPS), and right temporo-parietal junction (TPJ). The results of the category-selective contrasts are reported in Table 2.

**Table 2.** Category-sensitive brain regions

Category	Region	Cluster		Peak			
		*p_(FWE-cor)_*	Extent	*p_(FEW-cor)_*	*t*	*p_(unc)_*	*x*, *y*, *z* (MNI)
**People**	Left ATL	<0.001	699	<0.001	9.01	<0.001	−54, −10, −14
	Precuneus	<0.001	873	<0.001	7.48	<0.001	−4, −50, 28
	Right ATL	<0.001	297	0.001	5.87	<0.001	58, −6, −14
	vmPFC	<0.001	276	0.002	5.59	<0.001	−4, 58, −12
**Places**	Left PPA	<0.001	2597	<0.001	15.16	<0.001	−30, −40, −12
	Right RSC			<0.001	11.15	<0.001	10, −54, 10
	Left RSC			<0.001	11.09	<0.001	−10, −54, 8
	Left TOS	<0.001	1315	<0.001	12.59	<0.001	−38, −84, 32
	Right PPA	<0.001	617	<0.001	11.69	<0.001	28, −36, −16
	Right TOS	<0.001	872	<0.001	8.30	<0.001	40, −84, 34
	Left pMTG	<0.001	283	0.047	5.04	<0.001	−60, −58, −4
	Left aSTG	0.036	126	0.095	4.86	<0.001	−52, −6, −18
	Left dSFG	0.002	222	0.141	4.26	<0.001	−20, 22, 46
**Food**	Left OFC	<0.001	420	<0.001	8.47	<0.001	−22, 38, −16
	Left pITG	<0.001	940	<0.001	7.75	<0.001	−50, −52, −18
	Left IFG	<0.001	495	<0.001	7.20	<0.001	−42, 36, 10
	Right OFC	0.014	154	<0.001	6.80	<0.001	22, 38, −18
	Left preMotor	<0.001	331	0.010	5.39	<0.001	−44, 6, 24
	Left VTC	0.011	161	0.012	5.34	<0.001	−36, −28, −24
	Left Amygdala	0.018	146	0.623	4.28	<0.001	−20, −2, −24
	Right pMTG	0.014	155	0.742	4.18	<0.001	52, −48, −16
**Objects**	Left pMTG	0.026	136	0.685	4.23	<0.001	−54, −58, −2
**Animals**	Precuneus	<0.001	1685	<0.001	7.36	<0.001	4, −58, 30
	Right TPJ	<0.001	705	0.020	5.24	<0.001	48, −62, 20
	Left IPS	0.001	233	0.0.381	4.47	<0.001	−26, −56, 38
	Right SFG	0.014	155	0.0386	4.47	<0.001	28, 30, 56
	Left dlPFC	0.005	185	0.0901	4.03	<0.001	−38, 42, 38

*Note*. Significant clusters are reported separately for each semantic category (*p* < 0.05, cluster-corrected).

### The Role of Category-Selective Representations in Combined-Category Sentences

Having ascertained the presence of strong category-selective representation for single-category sentences, we asked whether these regions make a comparable contribution when concepts are combined across semantic domains. To this end, we assessed whether the activation patterns across this network produced by single-category sentences predict the neural response of the related combined-category sentences, or in other words, whether the response evoked by a sentence combining two categorical representations can be reconstructed from the individual contribution of the two categories.

ROIs were defined via an Omnibus ANOVA as isolated brain regions within which activity varies across single-category sentences without introducing bias towards a particular object category. Resulting ROIs were consistent with category-selective regions identified in the preceding section (see Figure S1 and Table S2 for network visualisation and ROI data).

As noted earlier, behavioural difference existed between the categories, which may partially account for the effects observed here. To ensure that category-selectivity effects were not due to RT confounds, the persistence of category effects after controlling for RT differences was assessed. Specifically, within ROIs described in the next section, for each subject, the beta responses for each category were regressed against the mean RT for each single-category condition. Then, the category-selective contrasts were recomputed on the residuals of this regression (now with the linear effects of RT removed). Category-selective responses persisted in all regions (*p* < 0.001) with the exception of left IPS, left lateral preMotor, vmPFC, and right lateral PFC. As we cannot be sure of the veracity of the category-selective nature of the responses in these regions, while we report them in Figure 2 and Figure S1, and Table 2 and Table S2, they have not been included in subsequent analyses.

**Figure 3.**
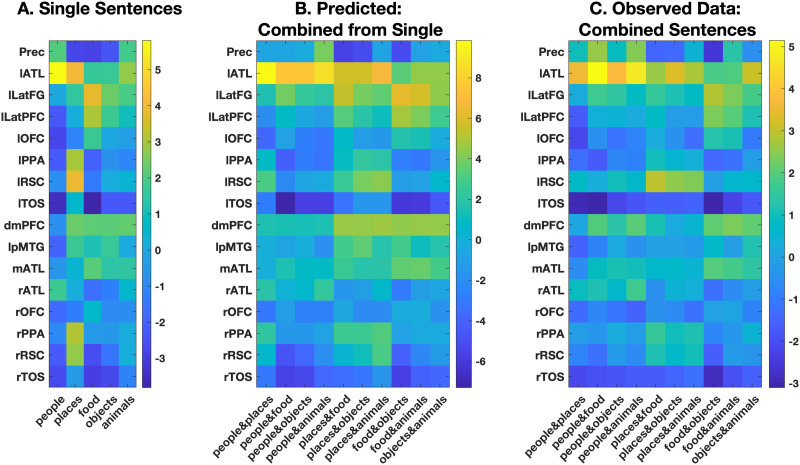
Consistency of regional response from single-category to combined-category sentences. Average beta values are shown for each selected ROI and sentence type. Values obtained from the single-category sentences (A) were averaged to create a prediction vector for the response to the sentence that combined those categories (B) for comparison against the veridical response to the combined-category sentences (C).

To construct the estimate of the combined-category sentences, within each ROI we took the response of single-category sentences (Figure 3A) and averaged them to form a prediction of the amplitude of the regional response for each combined-category sentence (Figure 3B). In this way, the regional response to sentences involving a person and place (Figure 3B, column 1) is predicted by the combination of the regional response of sentences about people and the regional response of sentences about places (Figure 3A, columns 1 and 2).

Overall, the response to the 10 combined-category sentences was closely predicted by their constituents. This is evident in the high congruence between the patterns evident in Figure 3B and Figure 3C. While the combined response to single-category sentences did not predict the response to combined-category sentences in dmPFC (*r* = −0.008, *p* = 0.98), pMTG (*r* = 0.50, *p* = 0.14), or left TOS (*r* = 0.59, *p* = 0.07), the combined response to single-category sentences did predict combined-category sentences in the remaining 13 regions. Prediction ability was high, with a mean *r* value across regions of 0.81 (min, 0.70, max, 0.92, all *p* values < 0.05). Thus, the combination of single-category sentences explained, on average, 66.0% of the observed response to combined-category sentences in these regions.

Importantly, RT differences in the single-category sentences did not predict response patterns in the combined-category sentences. Average RTs from the single categories used to form the predicted response (i.e., Figure 3B) were correlated with the RTs from the 10 combined-category sentences. Correlation was non-significant (*r* = −0.067, *p* = 0.86), indicating that this analysis is not contaminated by RT confounds.

To reconcile whole brain analysis showing a greater response for combined-category than single-category sentences in the precuneus and ROI analysis showing a category-selective effect in the precuneus, we performed two supplementary analyses. Firstly, we assessed the relationship between single-category and combined-category sentences in the category-selective precuneus ROI. The response in the category-selective precuneus ROI for combined-category sentences is super-additive. Specifically, the response for combined-category sentences is greater than the summed response to the composite single-category sentences (*t*(23) = 2.38, *p* = 0.026). This demonstrates that the category-selective defined precuneus ROI exhibited a greater response for combined- than single-category sentences, consistent with the whole brain contrast (c.f. Figure 1).

Next, within the precuneus cluster identified in the Combined-Category > Single-Category whole-brain analysis, we performed a correlation-based MVPA across voxels (see Materials and Methods). Specifically, the multivoxel pattern for each combined-category condition (e.g., People and Places) was reconstructed by combining the single-category sentences of its constituent categories (e.g., Single-Category People + Single-Category Places). Then, reconstructed patterns were used to distinguish between pairs of combined-category conditions (e.g., People and Places vs. Food and Places). The combination of single-category sentences could accurately predict the combined-category sentences in 36 out of 45 pairwise combinations (*p* < 0.05). Of the 9 cases where prediction failed, these were equally likely to occur when one of the two categories was present in both elements of the pair (6/30) as it was to occur when no categories were shared across the pair (3/15). These results indicate both the presence of category-sensitive neurons within this ROI and the consistency of category-sensitive patterns from single to combined sentences.

## DISCUSSION

Semantic knowledge involves not just the representation of single concepts but also the combination of these singular concepts into complex ideas. The construction of these higher-order units of meaning often requires the combination of concepts arising from different conceptual domains, which are differentially represented across the cortex. In this work, we asked two related questions: (1) Do specific brain regions play a particular role in combining concepts from different domains? (2) Do units of meaning that combine object categories continue to show a decentralised cortical representation, or are they represented in more centralised domain-general semantic regions?

To address these questions, we presented participants with meaningful sentences comprising concepts referring to a single semantic category (e.g., People: “The doctors treat the patients”) or different semantic categories (e.g., People and Places: “The employees go to the office”). We found that the precuneus showed an increased response when meaning had to be constructed across distinct semantic domains and that the distributed representation of conceptual contents across category-selective regions persists when multidomain, higher-order meaning is constructed.

### Precuneus Activity Increases When Concepts Are Combined Across Domains

The precuneus responded more strongly when sentences involved the combination of conceptual domains compared to when sentences involved a single semantic category. This activation remained significant when individual categories were iteratively removed from the contrast, indicating that the response is not driven by a specific category-selective response (Binder et al., 1999; Fairhall & Caramazza, 2013a). Furthermore, using the same univariate contrast, we did not observe any cortical modulation in other “language” regions associated with linguistic processing or complexity (Fedorenko, 2014; Santi & Grodzinsky, 2010), indicating that our strategy of matching sentence structure between single-category and combined-category items was successful and did not strongly influence brain regions associated with linguistic demand. Taken together, these findings suggest that the precuneus is a component of the neuronal circuitry involved in the flexible construction of unitary meaning originating from distinct conceptual domains.

Contemporary research has shown that the precuneus plays a dominant role in the general semantic system, being one of the most widely reported regions responding to semantically richer stimuli (Binder et al., 2009 for a review). The precuneus is a key element of the default mode network, an interconnected set of regions involved in internalised cognitive processes (Buckner et al., 2008; Buckner & DiNicola, 2019; Raichle et al., 2001). In this framework, the precuneus and posterior cingulate cortex have been described as a central hub of the network, acting as a convergence zone of distinct functional subsystems (Buckner et al., 2008; Xu et al., 2016). Concurrently, research has suggested that the precuneus plays a pivotal role in the core network enabling episodic memory retrieval and prospective imagining (Schacter et al., 2007; Schacter et al., 2012). It is interesting to note that within episodic memory retrieval the precuneus appears to play a linking role, flexibly binding together disparate conceptual information into meaningful units, a functional role that is notably consistent with the linking of concepts across domains to form transitory, higher-order semantic representations (for a related discussion, see Frankland & Greene, 2020).

These observations are not incompatible with previously proposed models, such as the “convergence zone hypothesis” (Damasio et al., 2004), the “distributed-plus-hub view” (Patterson et al., 2007), or the classical “hub-and-spoke” model (Rogers et al., 2004). According to the latter theories, a single hub in the ATL supports transmodal conceptual representation (Lambon Ralph et al., 2016). In this view, the function of the ATL is to link modality-specific information into a unitary representation related to a stable, singular, conceptual representation. The precuneus may play an analogous role, forming transitory links between discrete concepts from different domains into a higher-order unit of semantic meaning.

This interpretation is based on the assumption that concepts from two domains are combined when presented within these simple sentences, which is consistent with the automatic processioning of language (Kutas & Federmeier, 2011). However, it is possible that the precuneus response may reflect the presence of two domains not their combination. To exclude this possibility, it would be necessary to formulate a condition where concepts from different domains are present but are not integrated, which may not be possible due to the mind’s tendency to impose sense by linking even inexplicably connected concepts (e.g., “aardvark,” “cannon”). A potential indicator that concepts are being combined across domains is the superadditive nature of the precuneus response evident in the independently defined category-selective ROI—the response to combined sentences is greater than that of the summed response of the constituent single-category sentences. Such non-linearity of response has long been seen as a marker for integration in the multisensory literature (Holmes & Spence, 2005) and suggests some interaction beyond the representation of the two concepts in isolation in the present study.

The activation of the precuneus, together with the right AG, has been previously reported during the combination of noun–noun pairs (Graves et al., 2010). Converging findings from healthy adults and patients with neurodegenerative diseases (Price et al., 2015; Price et al., 2016) also support the role of the left AG in the integration of different concepts units (adjective–noun pairs) into meaningful combinations. While the general aim of these studies is consistent with the present work, here we adopted a paradigm with richer sentence stimuli (subjective, verb, complement) that was grounded in the combination of domain-specific concepts embedded in coherent meanings at the sentence level. Thus, while the AG may play a role in combining words to form more specific meaning (“lake house” or “red ball”), the precuneus is a potential mechanism by which concepts from different domains are generatively combined into higher-order meaning.

### Category-Selective Conceptual Representations for Single-Category Sentences

While often reported for image processing in ventral and dorsal streams, category-selective responses are less common for word stimuli (Bi et al., 2016). Here, using sentences depicting specific semantic categories, we observe robust category-selective responses for People, Places, Food and Animals.

Consistent with previous research, person-selective representations were seen in the precuneus and vmPFC, (Fairhall et al., 2014; Fairhall & Caramazza, 2013a; Leibenluft et al., 2004; Wang et al., 2016) as well as lateral ATL (Fairhall & Caramazza, 2013a; Grabowski et al., 2001; Tempini et al., 1998; Wang et al., 2016). Likewise, selective activation of bilateral PPA, TOS, and RSC for places is highly consistent not only with selectivity during the perception of places and scenes (Dilks et al., 2011; Epstein et al., 2007), but also in word-meaning related to places (Bi et al., 2016; Binder et al., 2009; Fairhall et al., 2014; Fairhall & Caramazza, 2013a), as well as spatially relevant geographic information about non-place objects such as food or people (Fairhall, 2020). Food-selective responses were found in bilateral OFC, consistent with previous literature investigating neural responses to food-related pictures (García-García et al., 2013; Killgore et al., 2003; Simmons et al., 2005), potentially reflecting the role of this region in processing the reward value (Mainen & Kepecs, 2009). Selective responses in VTC and amygdala have been previously reported in response to food pictures in relation to different motivational contexts (pre-/post-meal) in children and adolescents (Holsen et al., 2005). We did not observe a significant response in the left insula, which is frequently reported with studies using pictures (van der Laan et al., 2011, for a meta analysis). A food-selective response in the insula has been reported for word stimuli when participants access taste knowledge but the generalisation of this response to non-taste-related conceptual information is subtle and persists only at the voxel-level pattern (Fairhall, 2020). Animals selectively activated the precuneus, consistent with previous studies presenting participants with spoken names of animals (Binder et al., 1999). Responses in left IPS and right TPJ have been similarly reported by eliciting mental pictures of animals through spoken words (Lambert et al., 2002). We saw category selectivity for objects (here as human-made concrete items including manipulable objects) restricted to the pMTG, a region previously known to exhibit tool selectivity for word stimuli (Noppeney et al., 2006; Peelen et al., 2013).

### Combined-Category Representations Continue to Rely on Category-Selective Representations

Previous work investigating the combination of words has emphasised centralised, default mode, semantic systems (Graves et al., 2010; Palliera et al., 2011; for a discussion, see Frankland & Greene, 2020), elements of which are known to contain representations of different categories of objects (Bruffaerts et al., 2013; Devereux et al., 2013; Fairhall & Caramazza, 2013b; Liuzzi et al., 2020). To address whether the combination of concepts across domains continues to rely on distributed category-selective regions or is rather centralised into domain-general semantic systems, we compared the representation based upon single-category sentences to those of combined-category sentences across domain-sensitive ROIs. Specifically, we used the response evoked by the single-category sentences (e.g., People or Food) to predict the response to the relevant combined-category sentence (e.g., People and Food). The high consistency between the observed regional pattern of combined-category sentences and that predicted by combining the patterns of the relevant single-category sentences indicates that category-sensitive regions respond similarly when single-domain-specific information is processed and when a combination of domain-specific concepts are processed. Thus, both during the formation of complex ideas from single categories and in the combination of concepts across domains, distributed category-selective semantic representations continue to play a role in concept representation. This underscores the importance of distributed category-selective semantic representations both during the formation of complex ideas from single categories and in the combination of concepts across domains.

### Conclusions

Domain-specific concepts are a fundamental building block of our semantic cognition. At the same time, our cognitive system is constantly faced with the challenge of binding together distinct, category-selective semantic information in order to create the higher-order unitary meanings that allow flexible knowledge manipulation. In this work, we provide partial insight into how the human brain combines concepts into complex ideas. Our results suggest that the precuneus plays an important role in this regard, acting on diverse domain-specific semantic concepts across their respective neural representations and thus representing an important functional node of the human semantic system. Concurrently, the present findings showing highly comparable responses in category-sensitive regions, when both single and multiple domain-specific concepts are processed, indicates the persistence of decentralised representations of conceptual knowledge when derived information combining concepts from multiple categories are formed. Collectively, these results show the importance of category-selective representations in the formation of higher-order semantic representations and the potential role of the precuneus in binding these together.

## FUNDING INFORMATION

Scott Fairhall, H2020 European Research Council (StG), Award ID: 640594.

## AUTHOR CONTRIBUTIONS

Silvia Ubaldi*: Investigation: Lead; Project administration: Lead; Data curation: Equal; Formal analysis: Equal; Project administration: Lead. Giuseppe Rabini*: Data curation: Equal; Formal analysis: Equal; Writing – original draft: Equal; Writing – review & editing: Equal. **Scott L. Fairhall**: Conceptualization: Lead; Formal analysis: Equal; Writing – original draft: Equal; Writing – review & editing: Equal; Supervision: Lead; Funding acquisition: Lead. [*These authors contributed equally to the research.]

## TECHNICAL TERMS

Voxel: A three-dimensional pixel produced by volumetric imaging techniques such as fMRI.
Region of interest (ROI) analysis: As an alternative to investigating experimental voxels over the tens of thousands of voxels in the brain, ROI analysis is a more acute and focused measure that designates a specific region to investigate and in which to perform analysis.
Multivariate pattern analysis (MVPA): An information-based measure that, as applied to brain imaging techniques, tests whether the spatial pattern of brain activation reliably distinguishes between two cognitive states.
FWE cluster-corrected: A family-wise error correction to overcome multiple comparisons issues in fMRI using Random Field Theory. This approach considers the probability of observing a contiguous cluster of significant voxels, correcting for multiple comparisons over the whole brain volume.
