# Supplementary material for: Combining Concepts Across Categorical Domains: A Linking Role of the Precuneus
Source: Neurobiol Lang (Camb). 2021 Jul 13;2(3):354–71. doi: 10.1162/nol_a_00039 (PMC7611750; doi:10.1162/nol_a_00039)
Supplement: Supplementary file 1 [file nol-2-3-354-s001.docx]

**SUPPLEMENTARY MATERIAL**


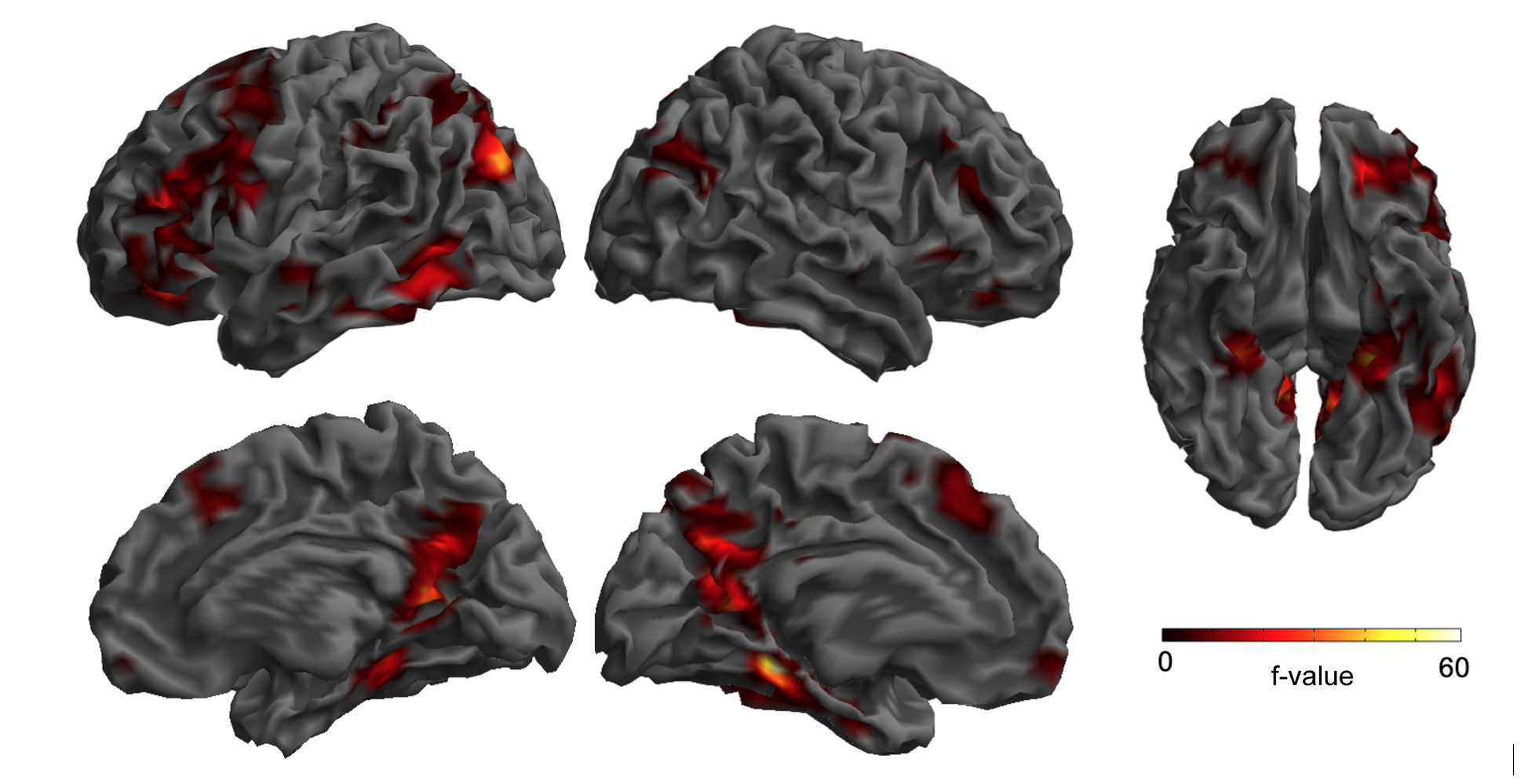


**Figure 1S.** Category-sensitive network. Regions of interest (ROIs) were defined through a whole brain Omnibus ANOVA, aimed to highlight brain regions showing category-preference while processing single-category sentences. A total of 19 ROIs were selected based on this contrast. ROIs were built creating a sphere (5mm radius) around the selected cluster peak, further combining it with the whole-brain cluster map deriving from the Omnibus ANOVA. Brain maps are displayed with a p<.001 threshold uncorrected.

**Table 1S. Regions of interest based on the whole brain Omnibus ANOVA**

| Region | Cluster | | Peak | | | |
| --- | --- | --- | --- | --- | --- | --- |
|  | ﻿p *_(FWE-cor)_* | Extend | p *_(FWE-cor)_* | F | p*_(unc)_* | *x,y,z*(MNI) |
| Left PPA | <.001 | 3031 | <.001 | 62.99 | <.001 | -30, -40, -12 |
| Left mATL |  |  |  | 13.27 |  | -30, -10, -32 |
| Left pMTG |  |  | <.001 | 23.89 | <.001 | -56, -60, -4 |
| Left latFG |  |  | <.001 | 22.88 | <.001 | -52, -50, -18 |
| Left TOS | <.001 | 3735 | <.001 | 45.97 | <.001 | -38, -84, 32 |
| Left IPS |  |  | <.001 | 19.55 | <.001 | -28, -74, 38 |
| Right RSC | <.001 | 3486 | <.001 | 38.71 | <.001 | 10, -54, 10 |
| Left RSC |  |  | <.001 | 33.37 | <.001 | -10, -54, 8 |
| Precuneus |  |  | <.001 | 29.90 | <.001 | -2, -62, 32 |
| Right PPA | <.001 | 489 | <.001 | 36.56 | <.001 | 28, -34, -16 |
| Left OFC | <.001 | 7328 | <.001 | 29.83 | <.001 | -24, 36, -16 |
| Left latPFC |  |  | <.001 | 27.66 | <.001 | -42, 36, 10 |
| Left preMotor |  |  | <.001 | 20.39 | <.001 | -24, 8, 56 |
| Right TOS | <.001 | 1211 | <.001 | 25.23 | <.001 | 44, -78, 36 |
| Left ATL | <.001 | 521 | <.001 | 24.44 | <.001 | -54, -10, -14 |
| Right ATL | <.001 | 271 | <.001 | 13.11 | <.001 | 58, -6, -16 |
| Right OFC | <.001 | 482 | <.001 | 12.87 | <.001 | 22, 38, -16 |
| vmPFC | <.001 | 217 | .007 | 10.37 | <.001 | -6, 58, -14 |
| Right latPFC | <.001 | 645 | .058 | 9.33 | <.001 | 44, 30, 22 |
| dmPFC | <.001 | 7320 | <.001 | 7.10 | <.001 | -4, 24, 44 |

Abbreviations: PPA: parahippocampal place area; mATL: medial anterior temporal lobe; pMTG: posterior middle temporal gyrus; ATL: anterior temporal lobe (lateral); TOS: transverse occipital sulcus; IPS: intraparietal sulcus; RSC: retrosplenial cortex; OFC: orbitofrontal cortex; latPFC: lateral prefrontal cortex; vmPFC: ventromedial prefrontal cortex; dmPFC: dorsomedial prefrontal cortex.


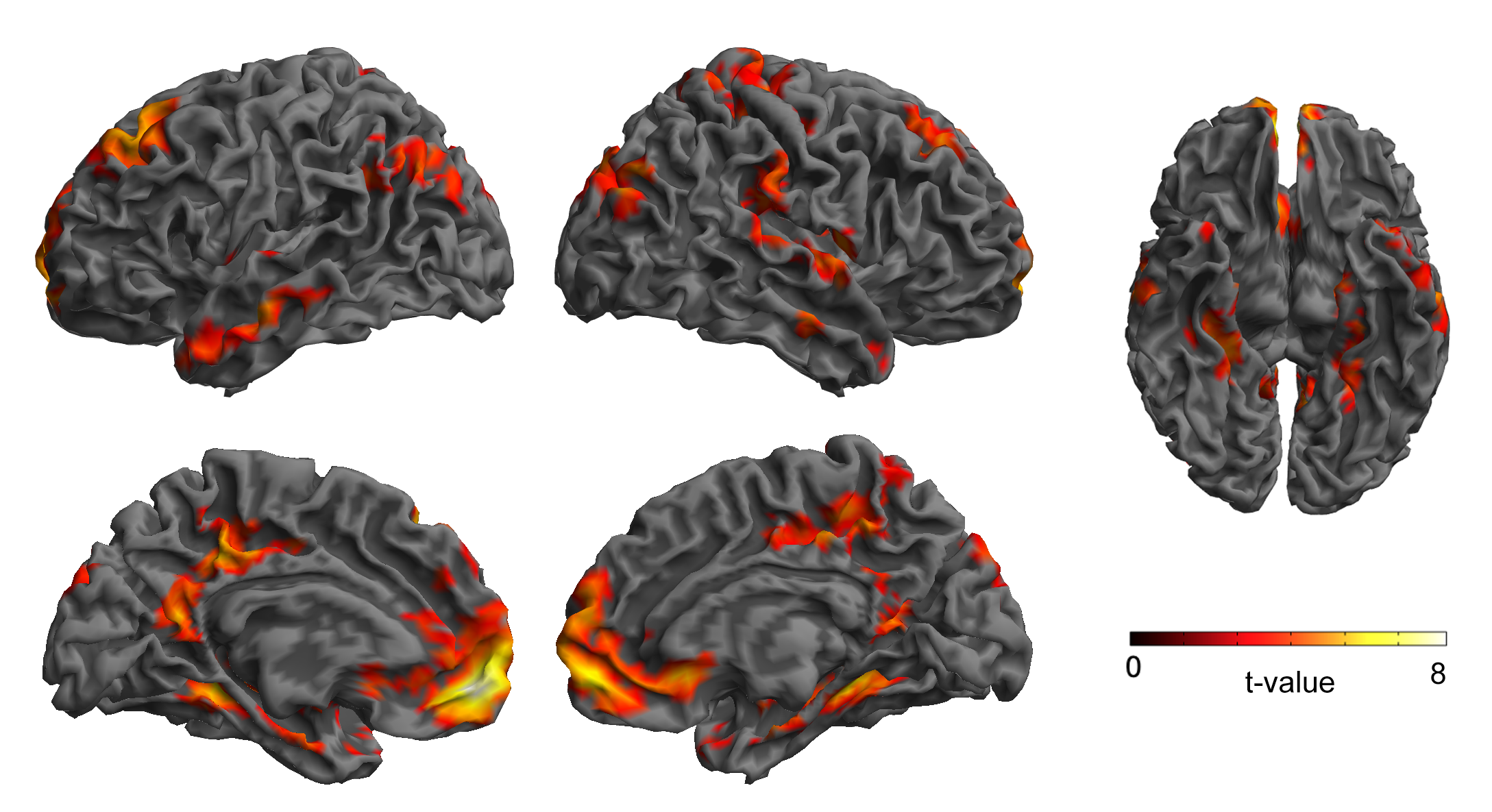
­­­

**Figure S2.** Meaningful>Bizarre Sentences. Overall difference between meaningful sentences (combined category and single category sentences) and bizarre sentences (p<.001 voxel, p<.05 fwe cluster corrected). See Table S2 for activation significance and locations.

**Table S2.** Significance, cluster extent and peak location for the contrast meaningful>bizarre sentences.

| Region | Cluster | | Peak | | | |
| --- | --- | --- | --- | --- | --- | --- |
|  | ﻿p *_(FWE-cor)_* | Extent | p *_(FEW-cor)_* | T | p*_(unc)_* | *x,y,z*(MNI) |
| vmPFC | <.001 | 5617 | <.001 | 8.88 | <.001 | -8 50 -10 |
| left PPA | <.001 | 1410 | <.001 | 7.02 | <.001 | -30 -40 -8 |
| right PPA | <.001 | 1549 | <.001 | 6.32 | <.001 | 32 -40 -10 |
| left SFG | <.001 | 1793 | <.001 | 6.22 | <.001 | -16 32 48 |
| Left TOS | <.001 | 1282 | <.001 | 6.11 | <.001 | 44 -80 30 |
| left ATL | <.001 | 864 | 0.001 | 5.96 | <.001 | -58 -12 -18 |
| PCC | <.001 | 4470 | 0.001 | 5.9 | <.001 | 0 -42 46 |
| right SFG | <.001 | 1018 | 0.002 | 5.75 | <.001 | 26 34 44 |
| left insula | 0.005 | 184 | 0.005 | 5.52 | <.001 | -46 -12 0 |
| right insula | <.001 | 1839 | 0.017 | 5.27 | <.001 | 54 -2 0 |
| left TOS | <.001 | 900 | 0.02 | 5.23 | <.001 | -50 -68 38 |
| right ATL | 0.001 | 233 | 0.027 | 5.17 | <.001 | 60 -12 -20 |
| left cuneus | 0.001 | 231 | 0.893 | 4.04 | <.001 | -12 -94 28 |

Abbreviations: vmPFC: ventromedial prefrontal cortex; PPA: parahippocampal place area; SFG: superior frontal gyrus; TOS: transverse occipital sulcus; ATL: anterior temporal lobe (lateral); PPC: posterior cingulate cortex.

**Table S3.** Full list of the 260 Italian-language stimuli used, with English translation.

| **Person-Person** |  |
| --- | --- |
| Le atlete si trovano con l’allenatore. | The athletes meet with the coach. |
| Gli anziani sono accompagnati dalle badanti. | The elderly are accompanied by caregivers. |
| Le commesse consigliano le acquirenti. | The salesmen advise buyers. |
| I fratelli proteggono le sorelle. | The brothers protect the sisters. |
| I dottori curano i pazienti. | The doctors treat the patients. |
| I poliziotti arrestano i ladri. | The police arrest the thieves. |
| I professori interrogano gli studenti. | The professors question the students. |
| Le maestre insegnano agli scolari. | The teachers teach the pupils. |
| Gli attori erano insieme al regista. | The actors were together with the director. |
| Il comandante istruisce i carabinieri. | The commander instructs the military police. |
| Il direttore premiava i dipendenti. | The manager rewarded the employees. |
| Le mamme sgridano i figli. | The mothers scold their children. |
| Le nonne coccolano i nipoti. | The grandmothers cuddle their grandchildren. |
| I fedeli si confessano coi sacerdoti. | The faithful confess to the priests. |
| I giudici assolvono gli imputati. | The judges acquit the accused. |
| Il sindaco salutava i cittadini. | The mayor greeted the citizens. |
| **Place-Place** |  |
| Le fabbriche sono fuori dalle città. | The factories are outside the cities. |
| Le palafitte illuminano i laghi. | The stilt houses illuminate the lakes. |
| Nelle scuole hanno costruito le aule. | In schools they built classrooms. |
| Con gli ascensori si salgono gli edifici. | With the lifts you go up the buildings. |
| I cimiteri sono dietro le chiese. | The cemeteries are behind the churches. |
| Dalle camere si accede ai bagni. | From the bedrooms one accesses the bathrooms. |
| Le baite sono in mezzo alla foresta. | The huts are in the middle of the forest. |
| Nei campeggi si montano le tende. | The tents are set up in the campsites. |
| Gli edifici si affacciano sulla piazza. | The buildings overlook the square. |
| Le cascate sono in cima alle montagne. | The waterfalls are on top of the mountains. |
| I casolari si ergono nella radura. | The cottages stand in the clearing. |
| Le scogliere emergono dal mare. | The cliffs emerge from the sea. |
| I ponti sono sopra le strade. | The bridges are above the roads. |
| Le oasi ristorano nel deserto. | The oases replenish the desert. |
| I grattacieli si vedono nelle metropoli. | Skyscrapers are found in metropolises. |
| La spiaggia arriva fino al bar. | The beach goes all the way to the bar. |
| **Food-Food** |  |
| I crauti accompagnano lo stinco. | Sauerkraut accompanies the pork knuckle. |
| La crostata si orna con le mandorle. | The tart is decorated with almonds. |
| Col limone si condisce l’insalata. | Salads are seasoned with lemon. |
| Dai gelati goccia la panna. | Cream drops from ice-creams. |
| I bignè sono ripieni di cioccolata. | The cream puffs are filled with chocolate. |
| Sulle frittelle si spalma la marmellata. | One spreads jam on the pancakes. |
| Le cotolette sono dentro ai panini. | The cutlets are inside the sandwiches. |
| Sulle torte si cola la glassa. | Icing is poured on cakes. |
| Le cipolle soffriggono con il sedano. | Onions are sautéed with celery. |
| Le polpette si cuociono con i piselli. | Meatballs are cooked with peas. |
| Le torte sono fatte con le uova. | The cakes are made with eggs. |
| Le mele sono accanto alle banane. | The apples are next to bananas. |
| La polenta si mangia coi funghi. | Polenta is eaten with mushrooms. |
| Lo spezzatino è fatto di carne. | The stew is made of meat. |
| Le more si raccolgono coi mirtilli. | Blackberries are harvested with blueberries. |
| Gli spinaci riempiono i ravioli. | Spinach fills the raviolis. |
| **Object-Object** |  |
| Le forchette sono accanto ai coltelli. | The forks are next to the knives. |
| Col gessetto si scrive sulla lavagna. | With chalk one writes on the blackboard. |
| Con l’accetta si taglia la legna. | The wood is cut with the hatchet. |
| Le pentole hanno tutte il coperchio. | The pots all have the lid. |
| I martelli percuotono le incudini. | The hammers strike the anvils. |
| Le cesoie tagliano come le forbici. | Shears cut like scissors. |
| Con la penna si scrive sui fogli. | With the pen one writes on the pages. |
| Le lampade illuminano i tavoli. | The lamps light up the tables. |
| Le finestre si rompono con le pietre. | The windows break with stones. |
| Gli specchi riflettono gli armadi. | The mirrors reflect the wardrobes. |
| Gli spazzolini sono vicino ai dentifrici. | The toothbrushes are next to the toothpastes. |
| Le gomme si trovano negli astucci. | The erasers are in the pencil cases. |
| I pettini si usano con le spazzole. | Combs are used with brushes. |
| Il portafogli può cadere dalla borsa. | The wallet can fall out of the bag. |
| Le borracce sono dotate di bicchieri. | The water bottles are equipped with cups. |
| I cucchiaini girano nelle tazze. | The spoons spin in the cups. |
| **Animal-Animal** |  |
| I cani rincorrono le lepri. | The dogs chase the hares. |
| I pipistrelli si nutrono di insetti. | Bats feed on insects. |
| I formichieri inseguono le formiche. | Anteaters chase ants. |
| Le volpi braccano le galline. | Foxes hunt hens. |
| I gatti cacciano gli uccellini. | Cats hunt baby birds. |
| Le aquile afferrano i topi. | Eagles grab mice. |
| I cerbiatti si nascondono dai lupi. | Fawns hide from wolves. |
| I delfini nuotano con le balene. | Dolphins swim with whales. |
| I cervi corrono insieme ai daini. | The red deer run together with the fallow deer. |
| Gli scimpanzé giocano con i gorilla. | The chimpanzees play with the gorillas. |
| Le giraffe bevono con le zebre. | The giraffes drink with the zebras. |
| I coccodrilli sono vicino alle antilopi. | The crocodiles are close to the antelopes. |
| Le capre dormono con le pecore. | The goats sleep with the sheep. |
| I cinghiali assomigliano ai maiali. | Wild boars resemble pigs. |
| I cavalli sono vicino ai puledri. | The horses are close to the foals. |
| I leoni sono insieme alle tigri. | The lions are together with tigers. |
| **Person-Place** |  |
| Gli studenti si trovano all’università. | The students are at the university. |
| I ladri sono chiusi in prigione. | The thieves are locked up in prison. |
| I bagnini si trovano sulla spiaggia. | Lifeguards are located on the beach. |
| Gli impiegati si recano in ufficio. | The employees go to the office. |
| Gli operai raggiungono i cantieri. | The workers reach the construction sites. |
| Le commesse sistemano i negozi. | The salesmen arrange the shops. |
| I calciatori corrono nel campo. | The footballers run into the field. |
| Gli sciatori scendono lungo le piste. | The skiers descend along the slopes. |
| I bidelli puliscono le scuole. | The janitors clean the schools. |
| I contadini coltivano anche gli orti. | The peasants also cultivate the vegetable gardens. |
| I bambini giocano nel parco. | Children play in the park. |
| I poliziotti lavorano alla centrale. | The cops work at the police station. |
| I dottori si trovano in ospedale. | The doctors are in the hospital. |
| Le modelle sono dentro l’atelier. | The models are inside the atelier. |
| I professori interrogano in aula. | The professors ask questions in the classroom. |
| I cacciatori si appostano nella foresta. | The hunters lurk in the forest. |
| **Person-Food** |  |
| l contadini sono accanto ai pomodori. | The farmers are beside to the tomatoes. |
| I bagnini mangiano i ghiaccioli. | The lifeguards eat popsicles. |
| Ai bambini piacciono anche i biscotti. | Kids also like cookies. |
| I poliziotti bevono il caffè. | The cops drink coffee. |
| Alle modelle piace molto l’insalata. | The models really like salads. |
| I cacciatori preparano lo spezzatino. | The hunters prepare the stew. |
| I pescatori puliscono i pesci. | Fishermen clean the fish. |
| Gli uomini si occupano della carne. | Men take care of the meat. |
| Agli anziani si prepara la minestra. | The soup is prepared for the elderly. |
| I cuochi cucinano la pasta. | The chefs cook the pasta. |
| I camerieri portano anche le pizze. | The waiters also bring the pizzas. |
| Alle donne può piacere lo yogurt. | Women may like yogurt. |
| Gli operai si preparano i panini. | The workers prepare the sandwiches. |
| Gli sportivi si portano i frullati. | Sportsmen bring smoothies. |
| I professori portano le banane. | The teachers bring the bananas. |
| Le mamme cuociono la crostata. | The mothers cook the tart. |
| **Person-Object** |  |
| I ladri si nascondono coi grimaldelli. | Thieves hide with lock picks. |
| I poliziotti impugnano le pistole. | The cops hold their guns. |
| I cacciatori imbracciano i fucili. | The hunters take up their rifles. |
| I contadini tagliano con le cesoie. | Peasants cut with shears. |
| Gli alpinisti utilizzano le borracce. | Mountaineers use water bottles. |
| I calciatori calciano i palloni. | The footballers kick the balls. |
| I professori sono accanto ai gessetti. | The professors are next to the chalks. |
| I bagnini usano i binocoli. | The lifeguards use binoculars. |
| Le commesse attaccano i cartellini. | The salesmen attach the cards. |
| I macellai tagliano con la mannaia. | Butchers cut with the cleaver. |
| I camerieri sistemano le forchette. | The waiters arrange the forks. |
| Gli impiegati scrivono al computer. | Employees write on the computer. |
| Gli operai battono coi martelli. | The workers strike with hammers. |
| I bidelli spazzano con la scopa. | Janitors sweep with brooms. |
| Gli attori sono davanti ai copioni. | The actors are in front of the scripts. |
| Gli stilisti utilizzano le forbici. | The stylists use scissors. |
| **Person-Animal** |  |
| I contadini allevano le galline. | Farmers raise chickens. |
| I cacciatori sparano ai cervi. | The hunters shoot the deer. |
| I fantini lavano bene i cavalli. | The jockeys wash well the horses. |
| I granchi pizzicano i bagnini. | The crabs pinch the lifeguards. |
| I cinghiali attaccano gli uomini. | Boars attack men. |
| I bambini giocano coi conigli. | Children play with rabbits. |
| Gli ornitologi studiano gli uccelli. | Ornithologists study birds. |
| I pescatori catturano i pesci. | Fishermen catch fish. |
| I cani abbaiano ai ladri. | Dogs bark at thieves. |
| Gli allevatori sono vicini alle mucche. | The farmers are close to the cows. |
| Le donne sono spaventate dagli insetti. | Women are scared of insects. |
| I gatti dormono con i padroni. | Cats sleep with their owners. |
| I turisti abbracciano i panda. | Tourists hug the pandas. |
| Gli spazzini spaventano i topi. | The scavengers frighten the mice. |
| Gli scoiattoli si avvicinano ai fotografi. | The squirrels approach the photographers. |
| I delfini sono insieme agli addestratori. | The dolphins are together with the trainers. |
| **Place-Food** |  |
| Le crostate si vendono al supermercato. | The tarts are sold in the supermarket. |
| Nelle spiagge si trovano i ghiaccioli. | On the beaches there are icicles. |
| Le pannocchie sono vendute nei parchi. | The cobs are sold in the parks. |
| In cucina si cuoce lo spezzatino. | The stew is cooked in the kitchen. |
| Negli orti crescono i pomodori. | Tomatoes grow in vegetable gardens |
| Negli uffici si mangiano i panini. | In the offices they eat sandwiches. |
| I caffè sono serviti al bar. | Coffees are served at the bar. |
| Nelle baite si griglia la carne. | Meat is grilled in the huts. |
| I salami sono appesi nelle cantine. | The salamis hang in the cellars. |
| Le banane sono coltivate nelle piantagioni. | Bananas are grown on plantations. |
| La pizza si può trovare nei ristoranti. | Pizza can be found in restaurants. |
| Le pere crescono nel frutteto. | Pears grow in the orchard. |
| I biscotti si comprano in pasticceria. | The biscuits are bought in the pastry shop. |
| Le mele rotolano sul prato. | Apples roll on the lawn. |
| La lattuga cresce nell’orto. | Lettuce grows in the garden. |
| I gelati possono cadere per strada. | Ice creams can fall on the street. |
| **Place-Object** |  |
| Le forbici si vendono al supermercato. | The scissors are sold in the supermarket. |
| Nel poligono si usano le pistole. | Guns are used on the shooting range. |
| Le matite si trovano negli uffici. | Pencils are found in the offices. |
| Le sdraio si trovano in spiaggia. | The deckchairs are located on the beach. |
| Nei teatri ci sono le sedie. | There are chairs in theatres. |
| Nei bagni possono esserci i pettini. | In the bathrooms there may be combs. |
| In montagna si beve con le borracce. | One drinks with water bottles In the mountains. |
| In cucina si utilizzano i coltelli. | Knives are used in the kitchen. |
| Nelle aule si può scrivere coi gessetti. | Chalk can be used to write in classrooms. |
| Le radici crescono nei sentieri. | The roots grow in the paths. |
| In fabbrica si usano i martelli. | Hammers are used in the factory. |
| Nelle palestre vengono usati gli asciugamani. | Towels are used in the gyms. |
| Con i fucili si spara nella foresta. | In the forest, one shoots with rifles. |
| Le cesoie si utilizzano negli orti. | Secateurs are used in vegetable gardens. |
| I tagliaerba si trovano nei giardini. | Lawnmowers are found in gardens. |
| I palloni rotolano sui prati. | The balloons roll over the lawns. |
| **Place-Animal** |  |
| I topi si nascondono nelle fogne. | Mice hide in the sewers. |
| I gatti vivono spesso dentro casa. | Cats often live indoors. |
| I delfini saltano nel mare. | Dolphins jump into the sea. |
| Nella foresta si trovano i lupi. | One finds wolves in the forest. |
| Nei giardini dormono i cani. | Dogs sleep in the gardens. |
| Le mucche possono vivere nelle stalle. | Cows can live in stables. |
| I gabbiani camminano sulle spiagge. | Seagulls walk on the beaches. |
| I cervi si vedono nei sentieri. | Deer are seen on the trails. |
| I cavalli si trovano nella scuderia. | The horses are in the stable. |
| I pipistrelli dormono nelle grotte. | Bats sleep in caves. |
| I piccioni svolazzano sulle piazze. | Pigeons flutter in the squares. |
| I conigli saltano negli orti. | Rabbits jump into the vegetable gardens. |
| Le tigri si trovano allo zoo. | Tigers are found in the zoo. |
| Gli scoiattoli si rincorrono nei parchi. | Squirrels chase each other in parks. |
| Le volpi possono essere nei boschi. | Foxes can be in the woods. |
| Le capre brucano nei campi. | Goats graze in the fields. |
| **Food-Object** |  |
| Il risotto si mangia con la forchetta. | Risotto is eaten with a fork. |
| I caffè si bevono nelle tazzine. | Coffee is drunk from cups. |
| I carciofi si lavano coi guanti. | The artichokes are washed with gloves. |
| Con le cesoie si stacca l’uva. | Grapes are detached with shears. |
| I pomodori si innaffiano con il tubo. | Tomatoes are watered with the hose. |
| Le lasagne sono preparate nelle teglie. | Lasagnas are prepared in baking sheets. |
| I cucchiaini servono per i dolci. | The spoons are for the desserts. |
| Le crostate cuociono nei forni. | The tarts bake in the ovens. |
| Col martello si rompe il cocco. | The coconut is broken with the hammer. |
| Le frittelle sono servite nei piatti. | Pancakes are served on plates. |
| Le cotolette sono fritte nelle padelle. | Cutlets are fried in frying pans. |
| I coltelli tagliano la carne. | Knives cut the meat. |
| Il latte si versa nei bicchieri. | Milk is poured into glasses. |
| L’arrosto si conserva nei tupperware. | The roast is preserved in tupperware. |
| La marmellata può sporcare la tovaglia. | Jam can dirty the tablecloth. |
| I pomodori sono dentro le ciotole. | The tomatoes are inside the bowls. |
| **Food-Animal** |  |
| I coccodrilli si nutrono di carne. | Crocodiles feed on meat. |
| Gli scoiattoli raccolgono i pinoli. | Squirrels collect pine nuts. |
| I pipistrelli amano molto la frutta. | Bats are very fond of fruit. |
| I gatti bevono sempre il latte. | Cats always drink milk. |
| I conigli rosicchiano la lattuga. | Rabbits gnaw on lettuce. |
| I cinghiali divorano i funghi. | Wild boars devour mushrooms. |
| I topi rubano il formaggio. | The mice steal the cheese. |
| Le capre mangiano anche le verdure. | Goats also eat vegetables. |
| I cani annusano le bistecche. | The dogs smell the steaks. |
| I delfini mangiano i pesci. | Dolphins eat fish. |
| Le mucche producono il latte. | The cows produce milk. |
| Ai cavalli piacciono le carote. | Horses like carrots. |
| I maiali sgranocchiano le ghiande. | Pigs munch on acorns. |
| Le volpi rubano le uova. | Foxes steal eggs. |
| Le galline beccano il mais. | The chickens peck at the corn. |
| I cervi mangiano tante bacche. | Deer eat lots of berries. |
| **Object-Animal** |  |
| Gli scoiattoli si trovano sulle panchine. | Squirrels are found on benches. |
| I merli si costruiscono i nidi. | The blackbirds build their nests. |
| I delfini lanciano i palloni. | Dolphins throw balloons. |
| Le gazze possono rubare le collane. | Magpies can steal necklaces. |
| Nelle reti cadono i pesci. | Fishes fall into nets. |
| I cani rosicchiano gli ossi. | Dogs gnaw on bones. |
| I lupi possono annusare le foto-trappole. | Wolves can smell photo traps. |
| I cervi saltano le radici. | Deer jump roots. |
| I cavalli sono accanto alle selle. | The horses are next to the saddles. |
| I topi evitano le trappole. | Mice avoid traps. |
| I leoni ruggiscono alla frusta. | Lions roar at the whip. |
| I koala abbracciano i rami. | Koalas hug the branches. |
| Le capre salgono sulla tettoia. | The goats climb onto the shed. |
| I gatti giocano coi gomitoli. | Cats play with balls of yarn. |
| I maiali mangiano nelle mangiatoie. | Pigs eat from feeders. |
| I criceti girano sempre nelle ruote. | Hamsters always spin in wheels. |
